# ACValidator: A novel assembly-based approach for *in silico* verification of circular RNAs

**DOI:** 10.1093/biomethods/bpaa010

**Published:** 2020-08-10

**Authors:** Shobana Sekar, Philipp Geiger, Jonathan Adkins, Erica Tassone, Geidy Serrano, Thomas G Beach, Winnie S Liang

**Affiliations:** b1 Neurogenomics Division, Translational Genomics Research Institute, Phoenix, AZ, USA; b2 Arizona Alzheimer’s Consortium, Phoenix, AZ, USA; b3 Banner Sun Health Research Institute, Sun City, AZ, USA

**Keywords:** CircRNAs, RNAseq, RNA expression

## Abstract

Circular RNAs (circRNAs) are evolutionarily conserved RNA species that are formed when exons “back-splice” to each other. Current computational algorithms to detect these back-splicing junctions produce divergent results, and hence there is a need for a method to distinguish true-positive circRNAs. To this end, we developed Assembly based CircRNA Validator (ACValidator) for *in silico* verification of circRNAs. ACValidator extracts reads from a user-defined window on either side of a circRNA junction and assembles them to generate contigs. These contigs are aligned against the circRNA sequence to find contigs spanning the back-spliced junction. When evaluated on simulated datasets, ACValidator achieved over ∼80% sensitivity on datasets with an average of 10 circRNA-supporting reads and with read lengths of at least 100 bp. In experimental datasets, ACValidator produced higher verification percentages for samples treated with ribonuclease R compared to nontreated samples. Our workflow is applicable to non-polyA-selected RNAseq datasets and can also be used as a candidate selection strategy for prioritizing experimental validations. All workflow scripts are freely accessible on our GitHub page https://github.com/tgen/ACValidator along with detailed instructions to set up and run ACValidator.

## Introduction

Circular RNAs (circRNAs) represent a large class of ubiquitously expressed noncoding RNAs that are formed when exons “back-splice” to each other. The advent of high-throughput RNA sequencing (RNAseq) technologies and bioinformatics algorithms has facilitated the identification of thousands of circRNAs in multiple cell and tissue types [1–4]. These studies have found that circRNAs are highly abundant and evolutionarily conserved, as well as exhibit cell type- and developmental stage-specific expression. CircRNAs are also more stable than linear RNAs since they are covalently closed loops without 5′/3′ termini or a polyadenylated tail. Furthermore, studies investigating their functional relevance have revealed that circRNAs can act asmicroRNA (miRNA) regulators [3, 5–7], decoys to RNA binding proteins [8], and regulators of parental gene transcription [9].

Several computational tools have been developed to identify these back-splicing events in RNAseq data. Strategies employed by these computational tools to identify circRNAs include: (i) a pseudo-reference-based strategy, which is used by the known and novel isoform explorer (KNIFE) tool [10] and (ii) a fragment-based strategy, which is used by find_circ [3], CIRCexplorer [11], Mapsplice [12], and the detect circRNAs from chimeric reads (DCC) tool [13]. While KNIFE constructs a pseudo-reference of all possible out-of-order exons to align reads against, fragment-based strategies detect circRNAs based on the mapping information of a split read’s alignment to the reference genome [14]. When segments of a split read align to the reference in a non-linear order, they are marked as potential circRNA candidates. Apart from these strategies, CircRNA Identifier(CIRI) uses concise idiosyncratic gapped alignment report (CIGAR) signatures in the alignment file to identify circRNAs [15].

Tool comparison studies have revealed that existing circRNA detection algorithms produce divergent results due to the use of different aligners, heuristics, and filtering criteria [16, 17]. Hence, there is a need for an *in silico* approach that can distinguish true versus false-positive circRNAs identified using these algorithms. To this end, we developed Assembly-based Circular RNA Validator (ACValidator), which can be used as an *in silico* verification strategy, as well as a candidate selection tool for experimental validation. While existing approaches focus on detection of circRNAs, our approach performs *in silico* verification of circRNAs detected using these existing approaches. ACValidator first extracts reads from a fixed window on either side of the circRNA junction of interest from the alignment file and assembles them to generate contigs. These contigs are then evaluated for alignment against the circRNA junction sequence. We defined four different stringency criteria, ranging from 10 to 60 base pairs (bps) overlap across the junction. When evaluated on simulated as well as experimental datasets, ACValidator achieves better performance in datasets with higher circRNA coverage compared to ones with lower coverage.

## Materials and methods

**Figure 1:**
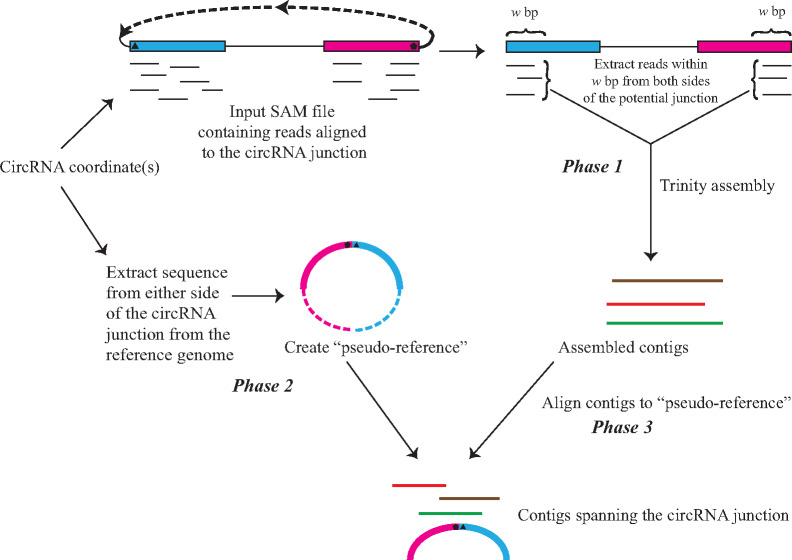
ACValidator workflow. ACValidator takes as input the SAM file and the circRNA junction coordinate(s) to be validated. In Phase 1, reads from either side of the junction within a user-defined window (*w*) are extracted and assembled using Trinity. A pseudo-reference containing the circRNA sequence of interest is generated from the reference genome in Phase 2. The pseudo-reference consists of *w* bp from either side of the circRNA junction of interest (solid blue and pink blocks in Phase 2). The broken blue and pink segments represent the remaining portions of the exons that constitute the circRNA but that are not a part of the pseudo-reference. Lastly, in Phase 3, the assembled contigs are aligned to this pseudo-reference and checked for overlap with the sequence of the junction to be validated.

ACValidator takes as input a sequence alignment mapping (SAM) file and the circRNA coordinate(s) to be validated (Fig. 1). ACValidator operates in three phases: (i) extraction and assembly of reads from the SAM file to generate contigs; (ii) generation of a pseudo-reference file; and (iii) alignment of contigs from Phase 1 against the pseudo-reference from Phase 2. First, reads are extracted from a user-defined window size *w* on either side of the given SAM file [(“start-coordinate”* *+* w*) and (“end-coordinate” − *w*); where the start-coordinate is the splice acceptor and the end-coordinate is the splice donor of the circRNA junction]. Our datasets were run using two different window sizes, where *w *=* *insert size or *w *=* *2* insert size, in order to understand the effect of window size on the results. We chose the above-mentioned window sizes in order to capture as many reads overlapping the circRNA junction as possible, given our use of paired-end sequencing data. However, a range of values between insert size and 2* insert size can be used, and users can adjust this parameter based on their library insert size. The tool thus extracts aligned reads within *w* bp on either side of the junction from the SAM file using SAMtools [18]. The extracted reads are then converted intoFASTQs and assembled using the Trinity assembler [19] to generate contigs (FASTA file). In the second phase, a pseudo-reference of the sequence surrounding the circRNA junction of interest is generated. This is performed by also extracting *w* bp from the end and start of the circRNA junction from the genome reference FASTA file (GRCh37; reference FASTA files of the genome of choice can be downloaded from https://genome.ucsc.edu/ or http://www.ensembl.org/) and concatenating the two sequences from end to end to capture the sequence on either side of the circRNA junction. Lastly, the assembled contigs from Phase 1 are aligned to the pseudo-reference from Phase 2 using the widely useed BWA-MEM [20] aligner. Each resulting alignment record is then examined to check whether it overlaps with the circRNA junction sequence using four different stringency criteria. The criteria require the following minimum lengths of alignment on both sides of the circRNA junction: high stringency—30 bp (total 60 bp overlap); medium stringency—20 bp (total 40 bp overlap); low stringency—10 bp (total 20 bp overlap); and very low stringency—5 bp (total 10 bp overlap). These stringency cut-offs were defined in order to capture as many tests as possible while still accounting for the extent of overlap between the contig and the circRNA junction sequence, as well as to assess whether we observe differences in sensitivity measurements across these cut-offs.

### Datasets used for evaluation

#### Simulated datasets

**Figure 2:**
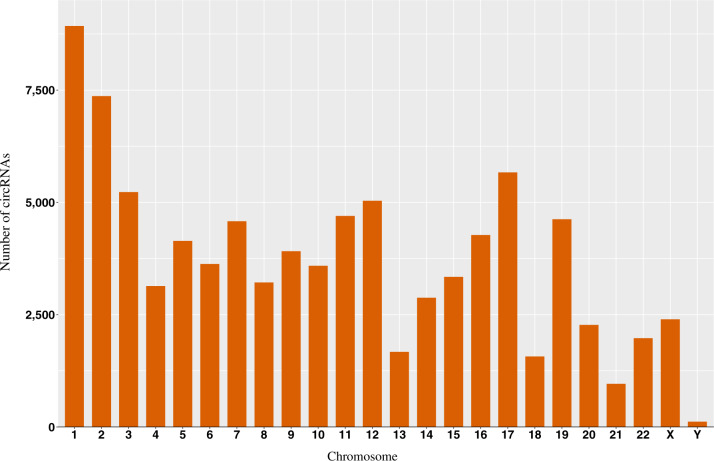
Chromosomal distribution of simulation datasets. Simulation datasets were generated using CIRI-simulator. The distribution of generated circRNAs across the different chromosomes is similar to the chromosomal size distribution.

We used CIRI-simulator [15] to generate 18 synthetic RNAseq datasets that had variable average number of supporting reads for circular and linear RNAs (2–80), as well as three different read lengths (50, 100, and 150 bp) to evaluate the performance of our workflow (Table 1). CIRI-simulator takes a FASTA-formatted reference file and a GTF annotation file as input, and generates circular and linear RNA sequences. Recently, Zeng *et al*. [17] re-designed this tool to generate synthetic reads for circRNAs deposited in circBase [21]. We generated simulated datasets with minimum circRNA size of at least 50 bp and insert size of 300 bp. Overall, an average of 89,293 circRNAs were generated across these simulated datasets. CIRI-simulator ensures this circRNAs map to locations distributed across the entire genome, thereby eliminating any bias associated with genomic location (Fig. 2). The generated true-positive simulation datasets are named using the convention pos_<circRNA_coverage>_<linearRNA_coverage>_ <read_length> (pos: positive; Table 1). Users can reproduce all simulated datasets by using the parameters described in Table 1 in CIRI-simulator.

**Table 1.** Simulation dataset parameters

Simulation set	Simulation dataset name	Average no. of circRNA supporting reads	Range of no. of circRNA supporting reads	Average no. of linear RNA supporting reads	Read length (bp)	No. of reads generated	Window lengths tested (bp)	Overlapping base thresholds tested (bp)	No. of candidates passing top 2% expression cut-off	No. of candidates below bottom 1% expression cut-off
1	pos_2_80_150	2	2–11	80	150	30** **926** **238	300, 600	60, 40, 20, 10	1196	13** **176
2	pos_2_80_100	2	2–10	80	100	46** **264** **524	300, 600	60, 40, 20, 10	1616	12** **829
3	pos_2_80_50	2	2–13	80	50	92** **271** **226	300, 600	60, 40, 20, 10	620	12** **670
4	pos_5_40_150	5	2–17	40	150	22** **725** **820	300, 600	60, 40, 20, 10	712	5261
5	pos_5_40_100	5	2–18	40	100	33 962 378	300, 600	60, 40, 20, 10	944	3887
6	pos_5_40_50	5	2–17	40	50	67 667 118	300, 600	60, 40, 20, 10	1296	4122
7	pos_10_20_150	10	3–28	20	150	24 867 386	300, 600	60, 40, 20, 10	1238	1391
8	pos_10_20_100	10	2–27	20	100	37 173 692	300, 600	60, 40, 20, 10	1628	1802
9	pos_10_20_50	10	2–26	20	50	74 092 068	300, 600	60, 40, 20, 10	1069	1641
10	pos_20_10_150	20	5–40	10	150	39 314 956	300, 600	60, 40, 20, 10	1456	1304
11	pos_20_10_100	20	4–42	10	100	58 844 010	300, 600	60, 40, 20, 10	1020	1039
12	pos_20_10_50	20	4–41	10	50	117 432 918	300, 600	60, 40, 20, 10	1405	1047
13	pos_40_5_150	40	8–65	5	150	73 285 586	300, 600	60, 40, 20, 10	1413	1303
14	pos_40_5_100	40	11–68	5	100	109 808 870	300, 600	60, 40, 20, 10	1775	1246
15	pos_40_5_50	40	10–74	5	50	219 344 762	300, 600	60, 40, 20, 10	1480	895
16	pos_80_2_150	80	15–119	2	150	143 598 648	300, 600	60, 40, 20, 10	1507	1115
17	pos_80_2_100	80	22–120	2	100	215 278 690	300, 600	60, 40, 20, 10	1746	897
18	pos_80_2_50	80	27–115	2	50	430 284 334	300, 600	60, 40, 20, 10	1493	960

All simulation datasets are based on data generated from human cerebellum and diencephalon, SH-SY5Y cells, Hs68 cells, HeLa cells, and HEK293 cells.

Columns C–F are user-defined parameters supplied to CIRI-simulator. Minimum circRNA size used in simulation: 50 bp, insert size used in simulation: 300 bp.

#### Experimental datasets

All subjects were enrolled in the Banner Sun Health Research Institute (BSHRI) Brain and Body Donation Program (BBDP) in Sun City, Arizona, and written informed consent for all aspects of the program, including tissue sharing, was obtained either from the subjects themselves prior to death or from their legally appointed representative. The protocol and consent for the BBDP were approved by the Western Institutional Review Board (Puyallap, Washington).

To test ACValidator on experimental data, we analyzed six pairs of ribonuclease R (RNase R)-treated and nontreated samples (*N* = 12; Table 2). RNase R is an exoribonuclease that selectively digests linear RNA but leaves behind circular structures, and it is hence widely used for circRNA enrichment. Three of these sample pairs were downloaded from the sequence read archive (SRA) [22] and were generated from HeLa and Hs68 cell lines treated or not treated with RNase R. The remaining three sample pairs were generated in-house from total RNA extracted from the middle temporal gyrus (MG) of three human postmortem healthy elderly control brains.

**Table 2.** Summary of experimental, non-simulated datasets used in this study

Data source	Dataset	Cell line/tissue type	RNase R treated?	Number of reads	Number of mapped reads	Median
						insert size
SRA	SRR1636985	HeLa	Yes	26** **619 ** **490	24** **370 ** **337	200
	SRR1637089	HeLa	No	89** **866** **900	63** **842** **205	140
	SRR1636986	HeLa	Yes	47** **011 ** **426	42** **027 ** **801	200
	SRR1637090	HeLa	No	71** **370** **620	53** **957** **685	130
	SRR444974	Hs68	Yes	316** **611 ** **710	271** **345 ** **091	160
	SRR444655	Hs68	No	314** **106** **316	109** **706** **923	150
In-house	MG_1	MTG	Yes	107** **609 ** **934	96** **300 ** **242	150
generated	MG_5	MTG	No	96** **215** **516	86** **315** **844	150
	MG_2	MTG	Yes	96** **840 ** **790	86** **560 ** **619	150
	MG_6	MTG	No	101** **609** **754	90** **750** **108	150
	MG_3	MTG	Yes	111** **576 ** **344	100** **264 ** **691	150
	MG_7	MTG	No	111** **314** **114	98** **894** **498	150

### Polymerase chain reaction validation

Polymerase chain reaction (PCR) was performed to experimentally validate the presence of selected circRNA candidates that were *in silico* validated by ACValidator. cDNA was synthesized from RNA isolated from the MG of the three tissue samples of interest using SuperScript II reverse transcriptase (ThermoFisher Scientific, Waltham, MA, USA). PCR was performed using 100 ng cDNA of each sample, 12.5 µl of Kapa HiFi polymerase (2X), 1 µl of the forward primer (5 µM), 1 µl of the reverse primer (5 µM), and 9.5 µl of molecular-grade water with the following thermocycler program: denaturation—60 s at 95°C, amplification—15 s at 95°C, 15 s at 55–63°C, 30 s at 72°C (cycle 35–40X), extension—60 s at 72°C. Primers were designed using Primer version 3 (http://bioinfo.ut.ee/primer3-0.4.0) and product sizes were assessed on a TapeStation 4200 instrument (Agilent Technologies, Santa Clara, CA, USA).

### Software requirements/dependencies

ACValidator can be implemented on a Linux-based high-performance computing cluster and has minimal requirements and dependencies. These requirements include the following: (i) Trinity version 2.3.1 or above; (ii) Python version 2.7.13 or higher with the pysam package installed; (iii) Bowtie2 version 2.3.0 [23] or above (required by Trinity); (iv) SAMtools version 1.4 or above and (v) BWA version 0.7.12 or above and (vi) BEDTools version 2.26 or above [24].

## Results and discussion

### Performance evaluation of ACValidator using simulated data

To evaluate ACValidator, we generated 18 simulation datasets with varying circular, linear RNA coverages, and read lengths (Table 1). We selected circRNA candidates above/below an expression cut-off as true positives/true negatives, respectively. For true-positive candidate set, we selected the top 2% of circRNAs based on supporting read counts (expression level), while for the true-negative candidate set, we selected the bottom 1% of circRNAs, also based on the supporting read counts (Supplementary Data, Tables S1 and S2). We replicated our analysis using two different window sizes: (i) *w* = insert size (300 bp) and (ii) *w *=* *2 * insert size (600 bp) (Materials and methods section).

We observed that simulations with higher circRNA coverages and longer read lengths achieved higher sensitivity (Supplementary Data, Fig. S1). Specifically, when using *w *=* *300 and overlap cut-off of 10 bp between the contig and pseudo-reference, simulations 7, 10, 13, and 16, which have average circRNA coverages >10 and read length of 150 bp, achieved over 81% sensitivity. For simulations 8, 11, 14, and 17, for which the average circRNA coverage was >10 but the read length was reduced to 100 bp, we observed a slight reduction in sensitivity by approximately 1–3%. However, when further reducing the read length to 50 bp, the sensitivity reduced to <=49% for simulations 9, 12, 15, and 18 (average circRNA coverage >10). When the average circRNA coverage was decreased to 5 or below (simulations 1–6), we observe an overall reduction in sensitivity while datasets with longer read lengths demonstrated improved performance compared to ones with shorter read lengths (20–26% sensitivity for simulations 3 and 6 and 57–79% for simulations 1 and 4, respectively). In datasets with lower circRNA coverage and/or shorter read length, this reduction in sensitivity was because Trinity did not find sufficient reads to assemble across these regions and hence was not able to generate contigs. We detect a similar pattern when using *w *=* *600 and did not observe a drastic difference in sensitivity between the two window sizes (Supplementary Data, Table S1). Furthermore, we calculated the F1 score [F1* *=* * (2*Precision* Sensitivity)/(Precision + Sensitivity)], a measure of accuracy, which indicates how well a tool achieves sensitivity and precision simultaneously (Supplementary Data, Fig. S1). We observed that for datasets with read length of 100 bp, the F1 score increases with coverage, while for datasets with read length of 150 bp, saturation is attained for datasets with average circRNA coverage >20. Overall, results from our simulation data indicate that higher circRNA coverage coupled with longer read length yields better performance of our approach.

**Figure 3:**
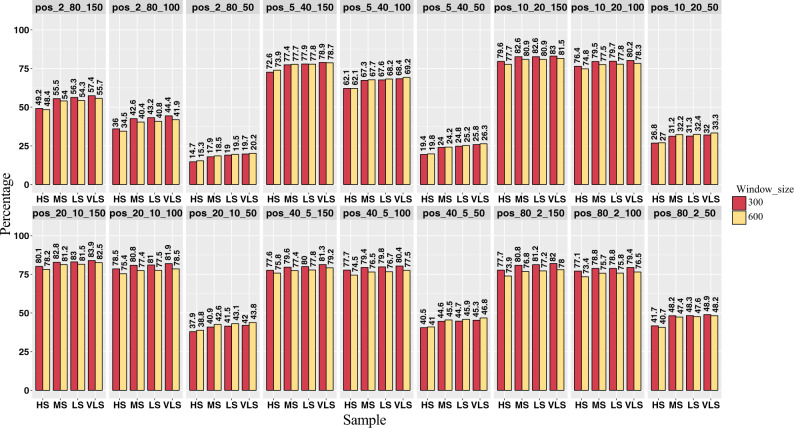
ACValidator performance across different overlap stringency thresholds in simulated datasets. Sensitivity measurements based on the top 2% of true-positive candidates were evaluated across four different overlap stringency thresholds (60, 40, 20, and 10 bp), as well as two different window sizes (300 and 600 bp). HS, high stringency; LS, low stringency; VLS, very low stringency.

Additionally, we evaluated sensitivity using four stringency thresholds for the number of overlapping bases (30, 20, 10, and 5 bp; Materials and methods section). We observed that for datasets with average circRNA coverage >10 and read lengths of 100 or 150 bp, there was no notable difference in the sensitivity across the different thresholds for the number of overlapping bases, with an average of 79–81% sensitivity, respectively (Fig. 3). However, for simulations with circRNA coverage <10 and read length of 50 bp, the sensitivity increases by ∼4–8% (10–34%) when reducing the overlap stringency from high to very low (Fig. 3). Thus, users can choose a less stringent overlap threshold such as 10 bp when running ACValidator on low coverage/short read length datasets but for higher circRNA coverage and longer read lengths, this threshold can be increased to 40 or 60 bp.

### Performance evaluation of ACValidator using experimental data

We next evaluated ACValidator using experimental, nonsimulated datasets generated from human tissues or cell lines (Table 2). Since we do not know the true-positive circRNAs for these datasets, we ran ACValidator on circRNAs that were called in both the RNase R-treated and nontreated datasets using six existing circRNA detection algorithms, find_circ version 1, CIRI version 2, Mapsplice version 2, KNIFE version 1.4, DCC version 0.4.4, and CIRCexplorer version 1.1.0. Each tool was run using RNAseq aligners and parameter settings as recommended by the respective developers. Co-ordinates returned by CIRI, MapSplice and DCC were converted from 1-based to 0-based coordinates to make them consistent with the other three detection tools. We considered those candidates that were called by at least three of the six tools (requiring exact co-ordinates to be detected by each tool) in both the treated and nontreated samples, and not depleted following RNase R enrichment, as true circRNAs (Supplementary Data, Table S3). A circRNA candidate is determined to be not depleted if the number of spliced reads per billion mappings [SRPBM; calculated as (number of circRNA supporting reads/total mapped reads)  × 10^9^] [2] does not decrease following enrichment. We thus ran ACValidator on these nondepleted potential true circRNA candidates for evaluation.

**Figure 4:**
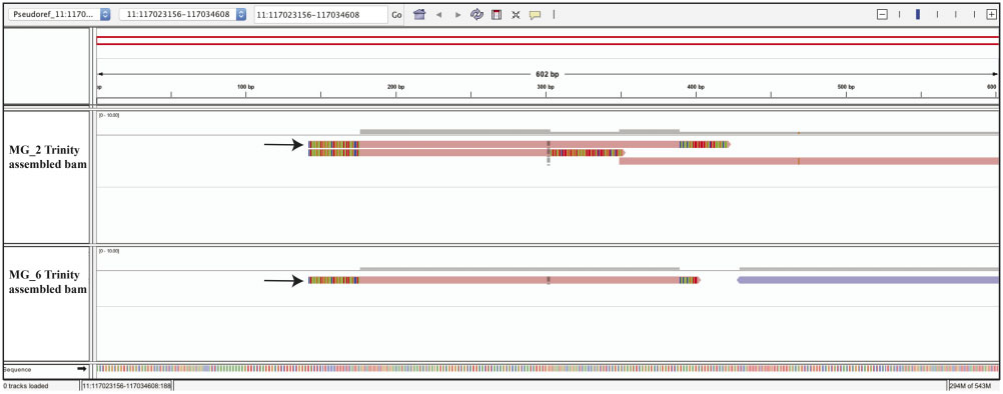
Integrated Genomics Viewer (IGV) screen shot of a circRNA candidate (chr11:117 023 156–117 034  608) Assembled contigs generated by ACValidator on RNase R-treated (top panel) and nontreated (bottom panel) human MG samples, aligned to the corresponding pseudo-reference. This circRNA was detected in both the treated and nontreated samples by at least three of the six existing circRNA prediction algorithms, and was not depleted following RNase R treatment. The circRNA junction of interest is at 300 bp, and pink bars that span over this junction represent the contigs that validate the junction (colored segments at the ends of contigs represent soft-clipped bases; arrows indicate the generated contigs that overlap with the circRNA junction). Reads from these samples aligned to the reference are shown in Supplementary Figure 3.

Overall, except for the SRR1636986–SRR1637090 pair, over 89% of the candidates that were called in both the treated and nontreated pairs were not depleted (i.e. SRPBM after RNase R treatment > SRPBM prior to treatment). Among these nondepleted candidates, ACValidator was able to construct contigs spanning the circRNA junction for >75% of them for the RNase R-treated samples and 47–57% of them for the nontreated samples using the medium-stringency criteria and both the window sizes (Table 3; Supplementary Data, Table S3 and Fig. S2). This increased verification rate for the treated samples is expected since RNase R treatment enriches for circRNAs and hence a higher number of back-splice junction supporting reads was observed. Furthermore, higher numbers of validated circRNAs were detected using lower stringency cut-offs for alignment overlap between contigs and junction sequences. As observed in the simulation datasets, the different window sizes did not notably affect the number of verifications among these experimental datasets (Table 3). Figure 4 shows an example of a circRNA [2, 25] that was validated by ACValidator in an MG-treated and nontreated pair (reads from this sample that align to the reference are shown in Supplementary Data, Fig. S3).

**Table 3.** Summary of ACValidator results on experimental datasets

Sample	No. overlap with pair	No. not depleted	Percent validated when *w* = insert size				Percent validated when *w* = 2* insert size			
			HS	MS	LS	VLS	HS	MS	LS	VLS
SRR1636985	1356	1243	73.53	78.52	79	79.57	73.45	78.84	79.57	80.13
SRR1637089	1356	1243	46.34	49.56	50.2	51.25	44.97	49.88	50.52	51.65
SRR1636986	780	594	79.46	84.68	85.35	86.03	80.81	86.36	87.21	87.71
SRR1637090	780	594	48.48	54.21	55.39	55.56	48.32	55.22	56.23	56.57
SRR444974	953	864	91.55	93.63	93.98	94.33	89.93	92.01	92.48	92.82
SRR444655	953	864	51.74	54.98	55.21	56.6	53.36	56.94	57.18	58.91
MG_1	1806	1691	72.8	77.05	77.94	78.83	71.67	77.35	78	78.71
MG_5	1806	1691	41.4	48.14	48.97	49.56	41.34	48.79	49.67	50.38
MG_2	1430	1331	71.9	76.86	77.46	78.14	69.72	76.11	76.63	77.31
MG_6	1430	1331	40.5	47.71	48.31	49.29	40.35	47.03	47.63	48.76
MG_3	1292	1148	71.95	76.74	77.53	78.48	70.38	75.87	76.83	77.7
MG_7	1,292	1148	44.08	51.48	52.26	53.4	44.51	51.39	52.18	53.57

In order to further evaluate the utility of our approach, we next compared the results from each individual tool to those from ACValidator. For this analysis, we used the top 100 most highly expressed candidates from among those we considered as true positives for these experimental datasets (called by at least three of six tools, in both treated and nontreated samples, and SRPBM after RNase R treatment > SRPBM before treatment; Supplementary Data, Table S3). Among the in-house treated MG samples, we observed that except for CIRCexplorer and Mapsplice, ACValidator was able to *in silico* validate a higher number of circRNAs, using the medium stringency (MS) criteria (20 bp overlap on either side of circRNA junction), than was detected individually by the other tools (Table 4). Among the SRA samples, however, our approach validated a fewer number of circRNAs than were detected by the individual tools except find_circ. Thus, results may vary depending on which individual tool is used for circRNA detection. Notably, the goal of ACValidator is to narrow down a list of potential high-confidence circRNAs and not for comprehensive and *de novo* detection of circRNAs.

**Table 4.** ACValidator and other tools' results on top 100 candidates from experimental datasets.

Sample	CIRI_count	CIRCexplorer_count	findCirc_count	Mapsplice_count	KNIFE_count	DCC_count	HS_count	MS_count	LS_count	VLS_count
SRR1636985	99	93	89	97	92	92	94	97	97	97
SRR1637089	99	92	87	97	92	92	74	78	79	79
SRR1636986	99	95	84	96	95	92	91	91	93	94
SRR1637090	95	88	57	85	95	83	71	77	78	78
SRR444974	99	97	91	97	95	95	97	98	98	98
SRR444655	99	97	76	38	95	90	84	84	84	84
MG_1	40	96	85	99	91	88	91	94	94	94
MG_5	55	93	72	98	91	80	77	83	83	83
MG_2	35	96	90	100	90	90	89	93	93	93
MG_6	50	92	68	98	90	82	75	81	81	81
MG_3	44	96	83	99	88	88	90	93	94	94
MG_7	40	94	79	99	88	85	84	88	91	91

The top 100 most highly expressed candidates were selected from the list of circRNAs called by at least three of six tools, in both treated and nontreated samples, and having SRPBM after RNase R treatment > SRPBM before treatment.

Each <tool_name>_count column lists the number of circRNAs among the top 100 detected by the tool. Similarly, HS, MS, LS, and VLS_count columns list the number of circRNAs among the top 100 that were validated by ACValidator using those stringency thresholds.

### Experimental validation of identified circRNAs

We performed experimental validations for six highly expressed circRNA candidates (average SRPBM > 650) that were *in silico* validated by ACValidator and that were detected by all six tools in each sample. Since we were interested in validating the presence of the circRNA and not their abundance, we performed PCR validations on these selected candidates. Each of our treated- and nontreated pair is generated from the same donor and hence, we ran validations on the non-RNase R-treated cDNA from each donor. For three of the candidates, ACValidator results were experimentally validated (Supplementary Data, Fig. S4A). For the candidate circRNA at chr10:116 879 948–116 931 050, ACValidator validated the circRNA junction in two of three samples, while chr9:113 734 352–113 735 838, chr5:38 523 520–38 530 768, and chr8:37 623 043–37 623 873 were validated in all three samples using all stringency cut-offs. For the remaining two candidates, we observed evidence of validation but because differently sized PCR products were generated, we could not determine the exact product size although it is possible that multiple circRNA species may be present. Additional PCR validations were performed on four circRNAs candidates demonstrating medium expression (average SRPBM > 300 and <600), that were validated by ACValidator, and that were called by at least three of six tools (Supplementary Data, Fig. S4B). These candidates include chr5:10 415 599–10 417 516, chr7:8 257 934–8 275 635, chr5:64 084 777–64 100 213, and chr4:56 277 780–56 284 152. All junctions were validated across the three untreated MG samples. PCR validations were also performed on two circRNAs candidates demonstrating low expression (average SRPBM < 90), that were validated by ACValidator, and that were called by three of six tools (Supplementary Data, Fig. S4C). These candidates include chr15:93 540 186–93 545 547 and chr3:3 178 943–3 186 394, and were validated across the three untreated MG samples. Sanger sequencing was also performed on the seven circRNAs candidates demonstrating medium or low expression, and although high background was observed for a large proportion of the reactions, three of the seven candidates were validated in at least one sample. These include junction 6 (for samples MG_1_5 and MG_3_7), junction 8 (for samples MG_2_6 and MG_3_7), and junction 9 (for sample MG_3_7).

### Computational cost overview

We ran our evaluations on a Linux-based high-performance computing cluster running CentOS version 7. As expected, the computational cost of our approach directly correlates with the number of reads in the input sample. The only rate-limiting step in using ACValidator is read alignment to generate the SAM file, which is performed prior to starting the workflow. The python script following this step requires <2 min of runtime for an input SAM file of **∼**8 GB, thus making our approach highly computationally efficient.

### Future directions

We present ACValidator, a novel bioinformatics workflow, which can be used to validate circRNA candidates of interest *in silico* and thus helps to identify true-positive candidates. This workflow is applicable to non-polyA-selected RNAseq datasets and can be used to validate circRNAs from various sample types and diseases. When different circRNA detection algorithms identify different circRNA candidates, ACValidator can be used as a complementary/orthogonal strategy to narrow down specific candidates of interest and thus serve as a circRNA candidate prioritization tool for experimental validations or functional studies.

When evaluated on simulated datasets, ACValidator demonstrates improved performance when higher numbers of circRNA supporting reads are available along with longer read lengths of the sequencing library. Thus, coverage as well as read length are important factors contributing to the performance of ACValidator, since a higher number of reads as well as longer read lengths result in improved assembly. When tested on circRNA candidates that were not depleted between RNase R-treated and nontreated sample pairs, we observed a higher verification rate in treated samples, as expected, since those samples are enriched for circRNAs. Window size, the region from where reads are extracted for assembly, is an important parameter for our approach. Through testing of two different window sizes, one equal to and one twice the insert size, we did not observe notable differences in the number of verifications. Additionally, we applied different stringency thresholds based on the extent of contig alignment, but we did not observe notable differences in the number of validated candidates across the different thresholds for highly expressed candidates.

Although our workflow provides a novel approach for *in silico* verification, we are limited by a few caveats. Primarily, since our assembly analysis relies on reads that extend across circRNA junctions, we are limited in our ability to *in silico* validate circRNAs whose expression may be low, especially in samples that are not enriched for circRNAs. Second, since we are limited by the lack of a gold standard circRNA reference dataset, we rely on simulation datasets for evaluation of our approach, which is based on informatically predicted circRNAs detected by various studies and deposited in circBase. Further, since we do not know the true-positive events in our experimental datasets, we evaluated candidates that are not depleted by RNase R. However, it is still not known whether RNase R treatment introduces any bias in circRNA detection, especially since some circRNAs are sensitive to RNase R [2, 11, 26, 27].

Future versions of ACValidator will include contig alignment visualization options built into the workflow, as well as alternative strategies to generate contigs for circRNAs with lower expression levels. As circRNAs continue to gain attention as an interesting class of noncoding RNAs, development of novel approaches, including implementation of statistical tests to estimate false discovery rates in circRNA detection, is needed. Continued progress in improving our understanding of the biology of circRNAs will be necessary for such algorithmic development. These findings will be crucial not only for functional analysis, but also for the development of more accurate circRNA detection algorithms.

## Data and tool availability

All data generated through this study are accessible through the European Genome Archive (EGA; accession EGAS00001003128). ACValidator is freely available on our GitHub page: https://github.com/tgen/ACValidator, along with detailed instructions to set up and run the tool (also available as Supplementary Text 1). ACV_launcher.sh, a wrapper/launcher script, is also included to enable verification of multiple co-ordinates.

## Supplementary Material

bpaa010_Supplementary_Data
