# Supplementary material for: ACValidator: A novel assembly-based approach for in silico verification of circular RNAs
Source: Biol Methods Protoc. 2020 Aug 10;5(1):bpaa010. doi: 10.1093/biomethods/bpaa010 (PMC7415914; doi:10.1093/biomethods/bpaa010)
Supplement: bpaa010_Supplementary_Data [file bpaa010_supplementary_data.zip › S1_Fig_v2.pdf]

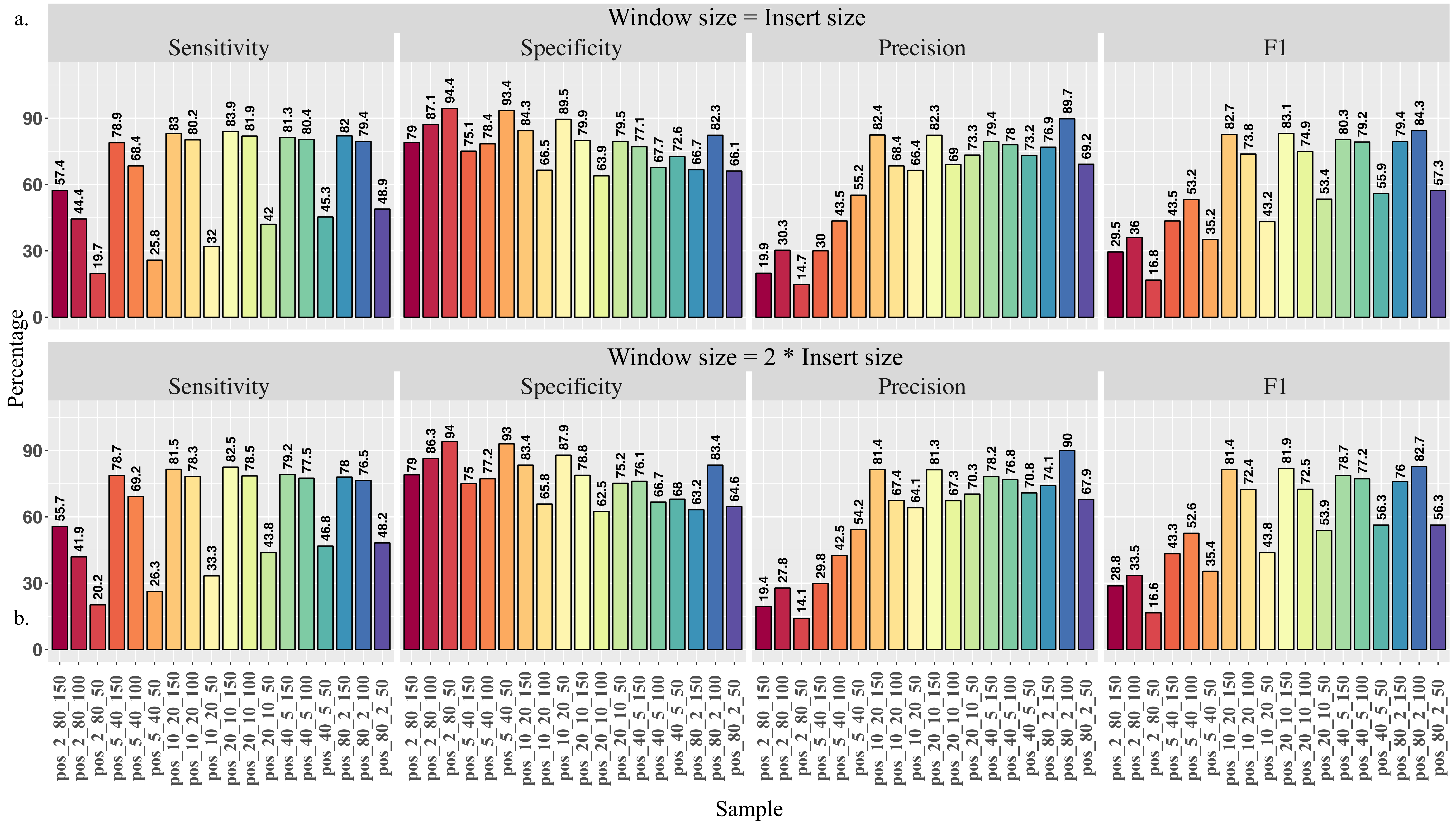

a.
Window size = Insert size
Sensitivity
Specificity
Precision
F1
b.
Window size = 2 * Insert size
Percentage
Sample
pos_2_80_150
pos_2_80_100
pos_2_80_50
pos_5_40_150
pos_5_40_100
pos_5_40_50
pos_10_20_150
pos_10_20_100
pos_10_20_50
pos_20_10_150
pos_20_10_100
pos_20_10_50
pos_40_5_150
pos_40_5_100
pos_40_5_50
pos_80_2_150
pos_80_2_100
pos_80_2_50

**Supplementary Figure 1**. ACValidator performance on true positive and true negative circRNA candidates from simulated datasets. The top 2% of circRNA candidates based on the number of supporting reads were considered true positives (TP) and the bottom 1% of circRNA candidates based on the number of supporting reads were considered true negatives (TN). The simulation datasets are described in Table 1 and each simulation dataset (x-axis) is named using the following naming convention: pos_<circRNA_coverage>_<linearRNA_coverage>_ <read_length> (pos: positive). The panels represent ACValidator performance on TP and TN candidates when using an overlap cut-off of 10 bp between the contig and pseudo-reference, and a) window size = insert size (300 bp) and b) window size = 2 * insert size (600 bp).
P =TP/(TP+FP); S = TP/(TP + FN); Sp = TN/(TN+FP); F1 = (2 * P * S)/ (P + S), FP, false positives; FN, false negatives; P, precision; S, sensitivity; Sp, specificity.
