# Supplementary material for: ACValidator: A novel assembly-based approach for in silico verification of circular RNAs
Source: Biol Methods Protoc. 2020 Aug 10;5(1):bpaa010. doi: 10.1093/biomethods/bpaa010 (PMC7415914; doi:10.1093/biomethods/bpaa010)
Supplement: bpaa010_Supplementary_Data [file bpaa010_supplementary_data.zip › S2_Fig_v2.pdf]

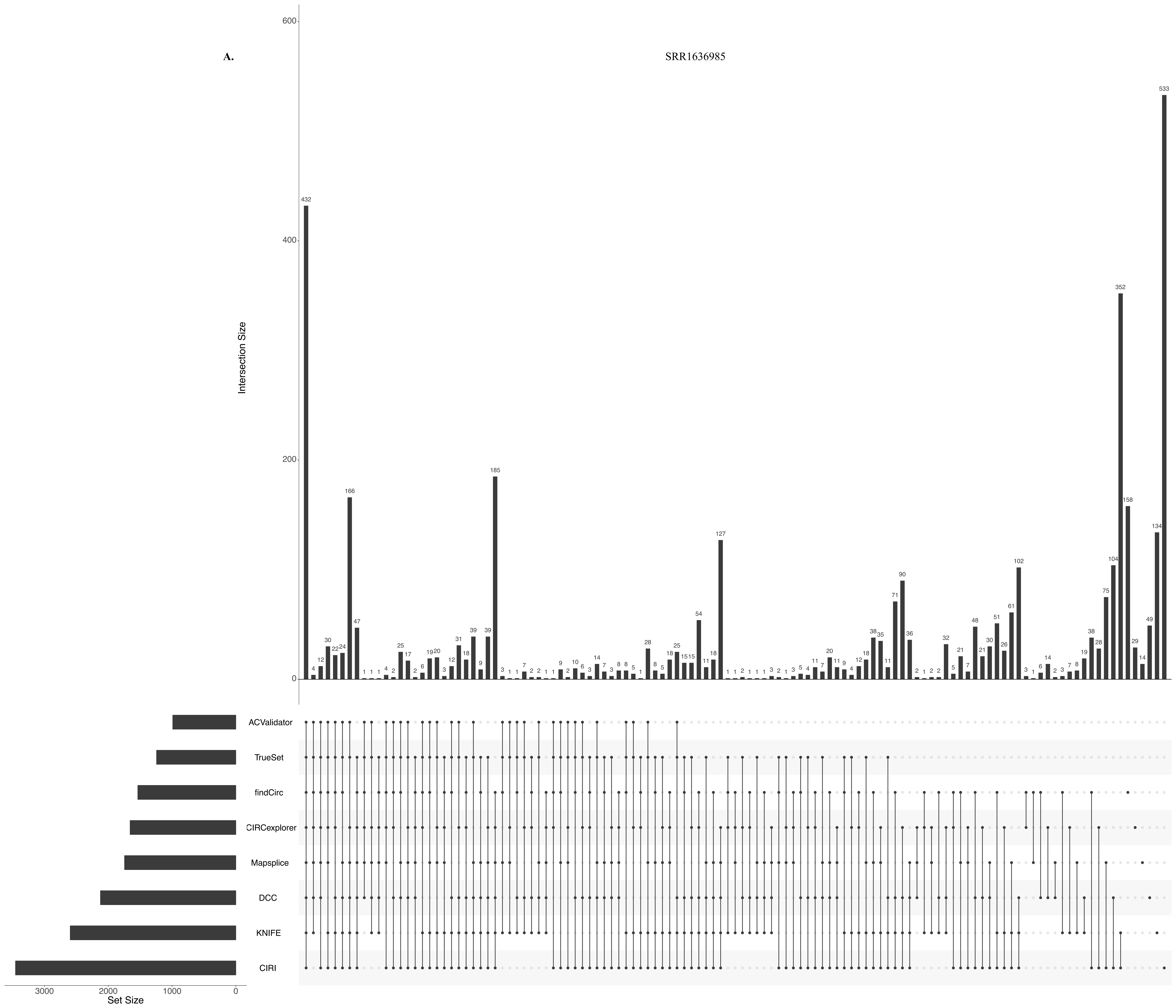

A.
SRR1636985
Intersection Size
Set Size
ACValidator
TrueSet
findCirc
CIRCexplorer
Mapsplice
DCC
KNIFE
CIRI

B.

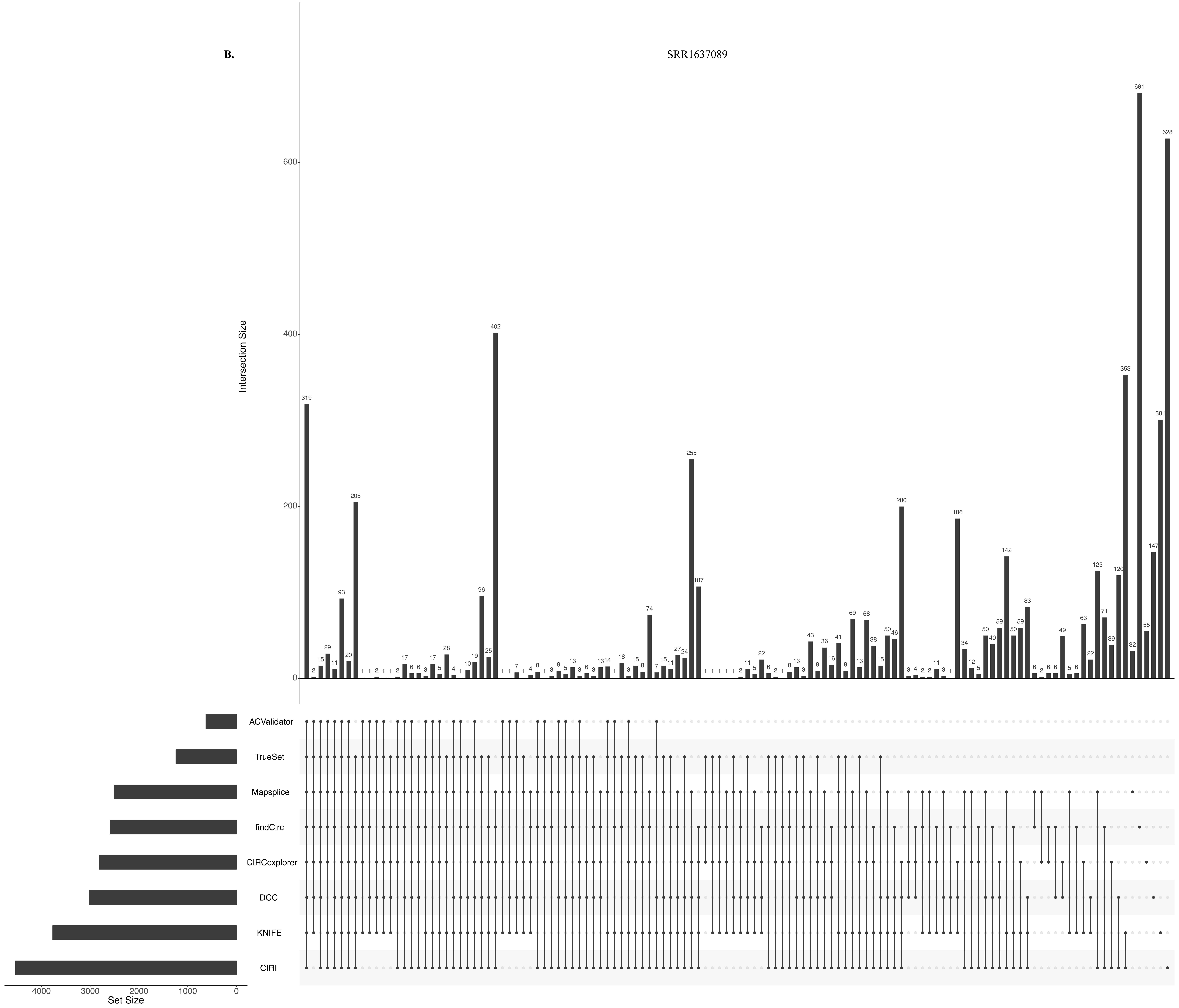
SRR1637089
Intersection Size
ACValidator
TrueSet
Mapsplice
findCirc
CIRCexplorer
DCC
KNIFE
CIRI
Set Size

C.

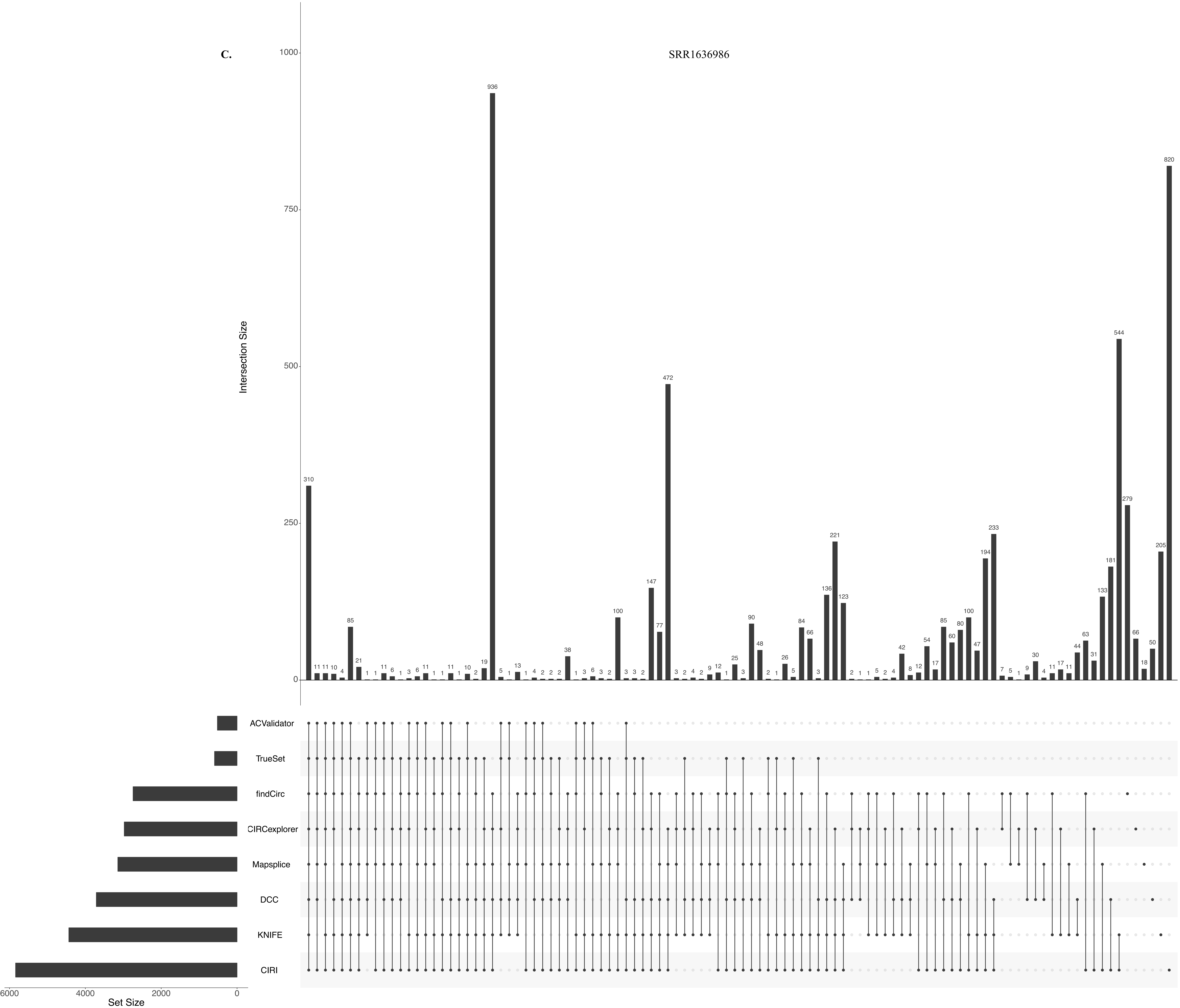
SRR1636986
Intersection Size
ACValidator
TrueSet
findCirc
CIRCexplorer
Mapsplice
DCC
KNIFE
CIRI
Set Size

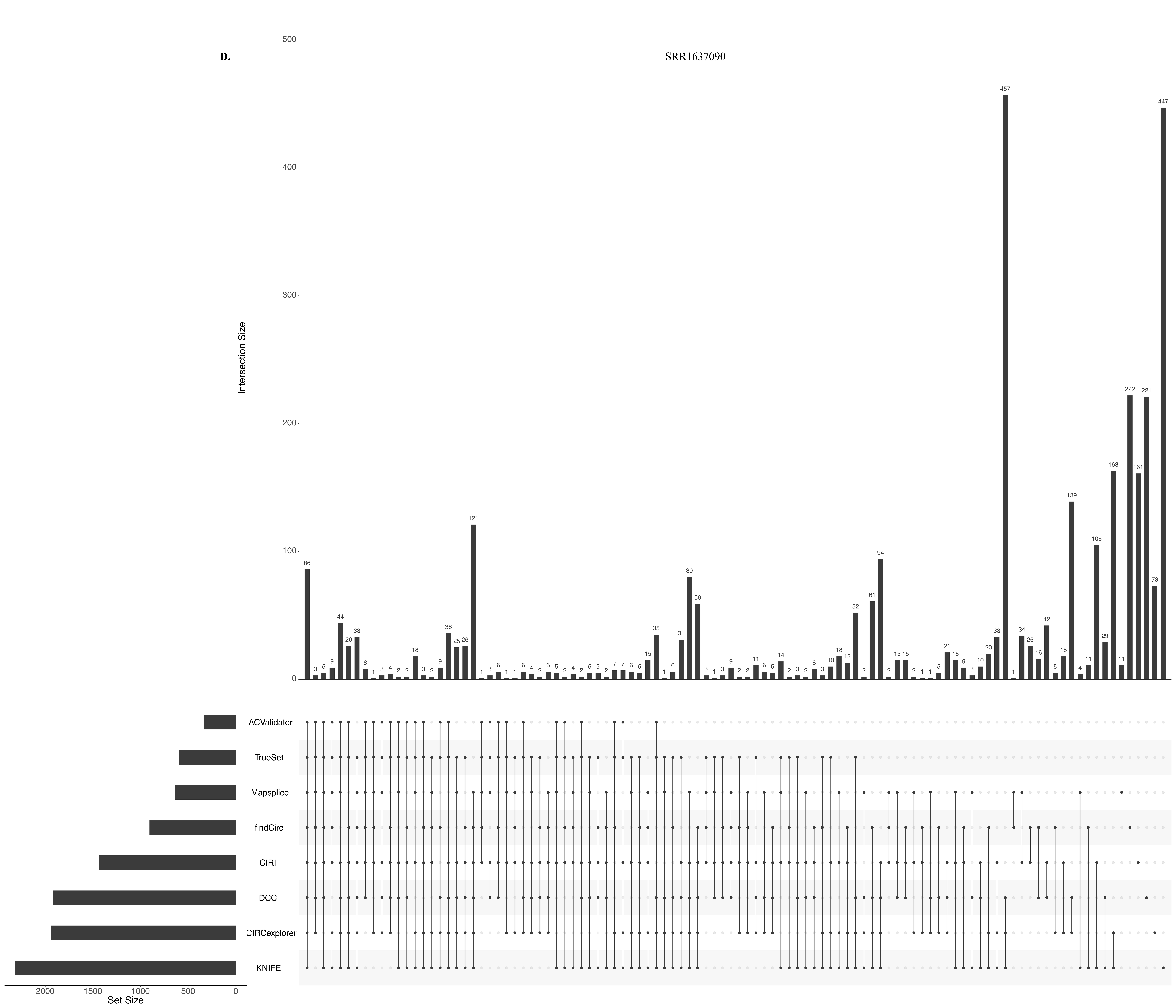
D.
SRR1637090
Intersection Size
ACValidator
TrueSet
Mapsplice
findCirc
CIRI
DCC
CIRCexplorer
KNIFE
Set Size

E.

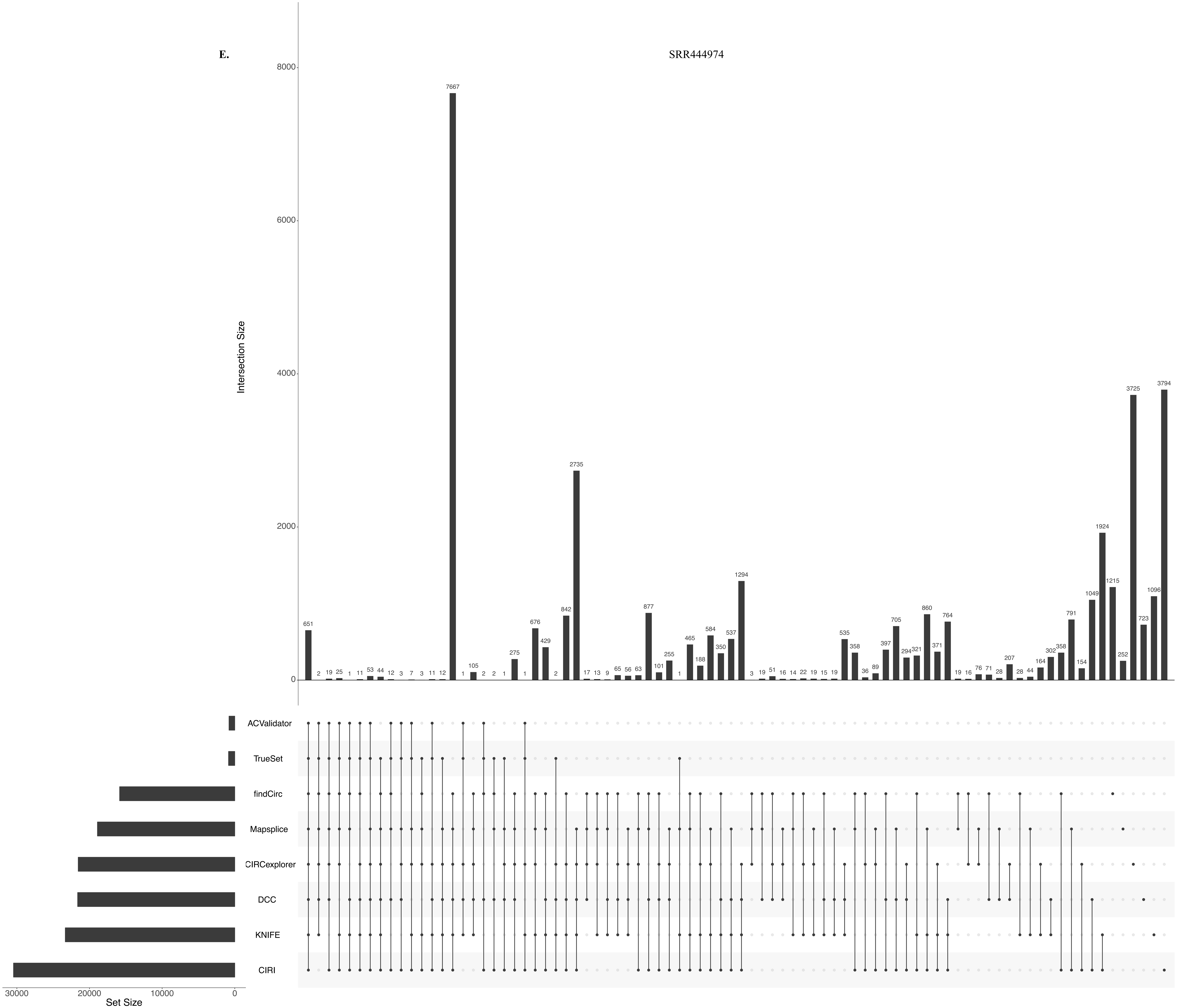
SRR444974
Intersection Size
Set Size
ACValidator
TrueSet
findCirc
Mapsplice
CIRCexplorer
DCC
KNIFE
CIRI

F.

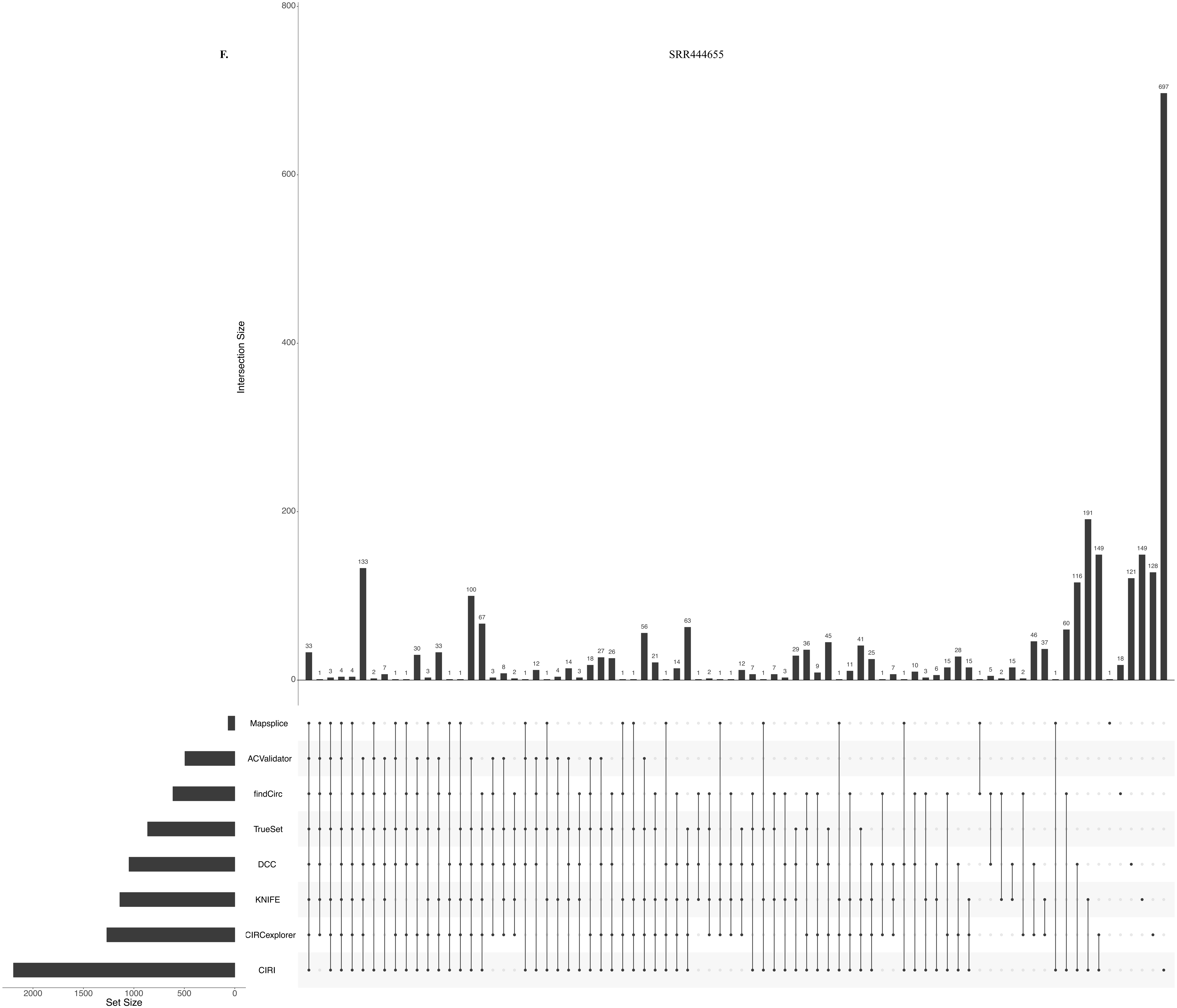

SRR444655
Intersection Size
Set Size
Mapsplice
ACValidator
findCirc
TrueSet
DCC
KNIFE
CIRCexplorer
CIRI

G.

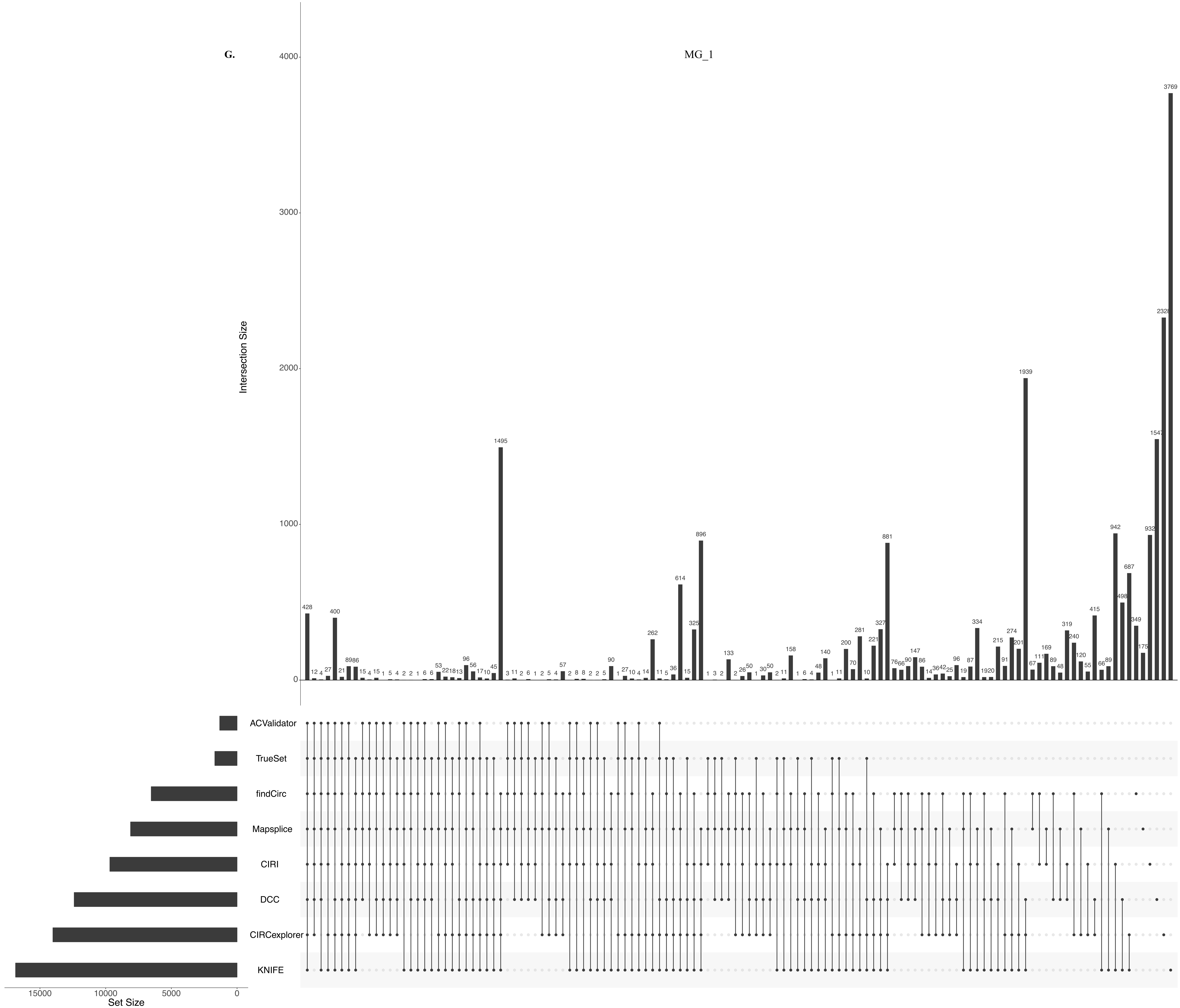

MG_1
Intersection Size
0
1000
2000
3000
4000
ACValidator
TrueSet
findCirc
Mapsplice
CIRI
DCC
CIRCexplorer
KNIFE
Set Size
15000
10000
5000
0

H.

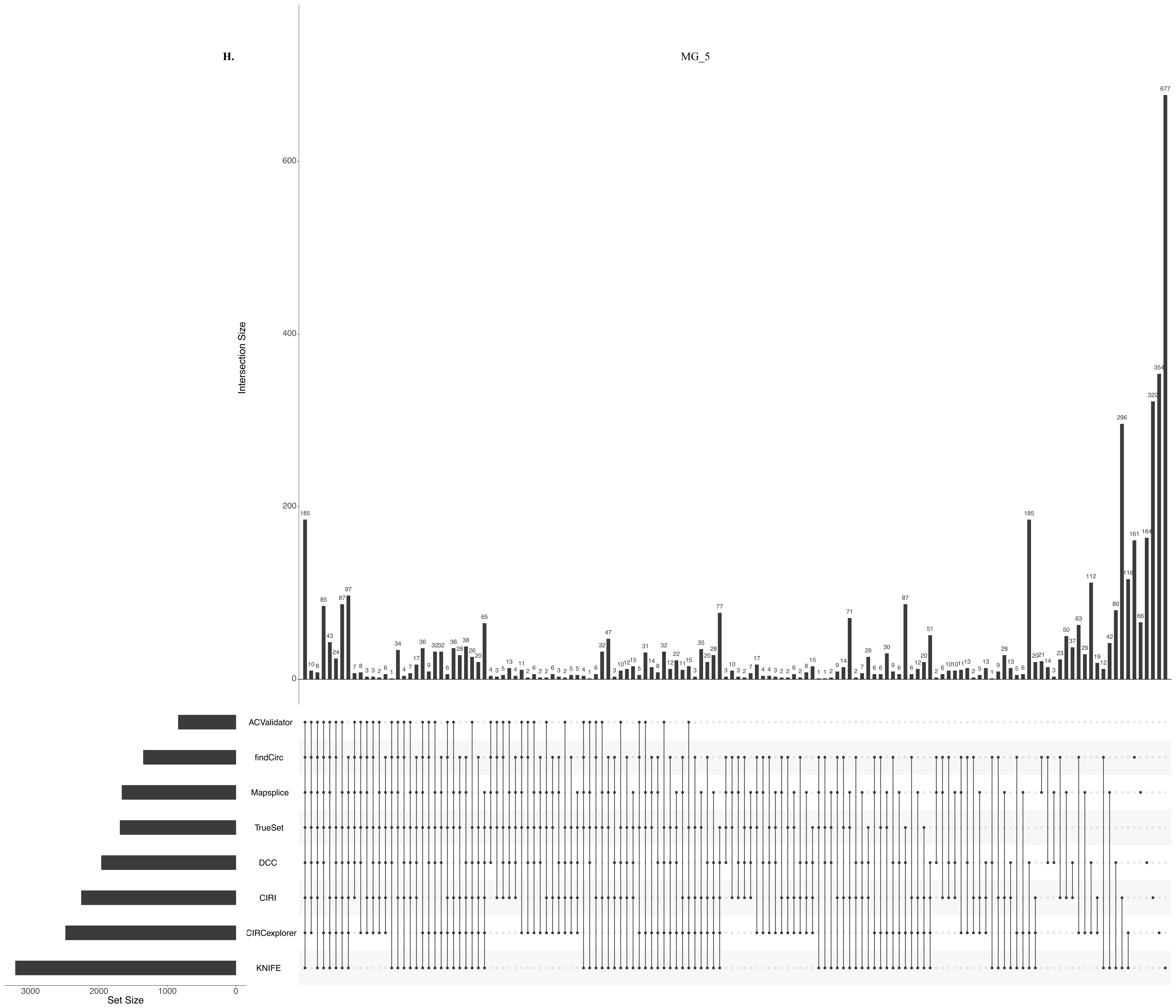
MG_5
Intersection Size
Set Size
ACValidator
findCirc
Mapsplice
TrueSet
DCC
CIRI
CIRCexplorer
KNIFE

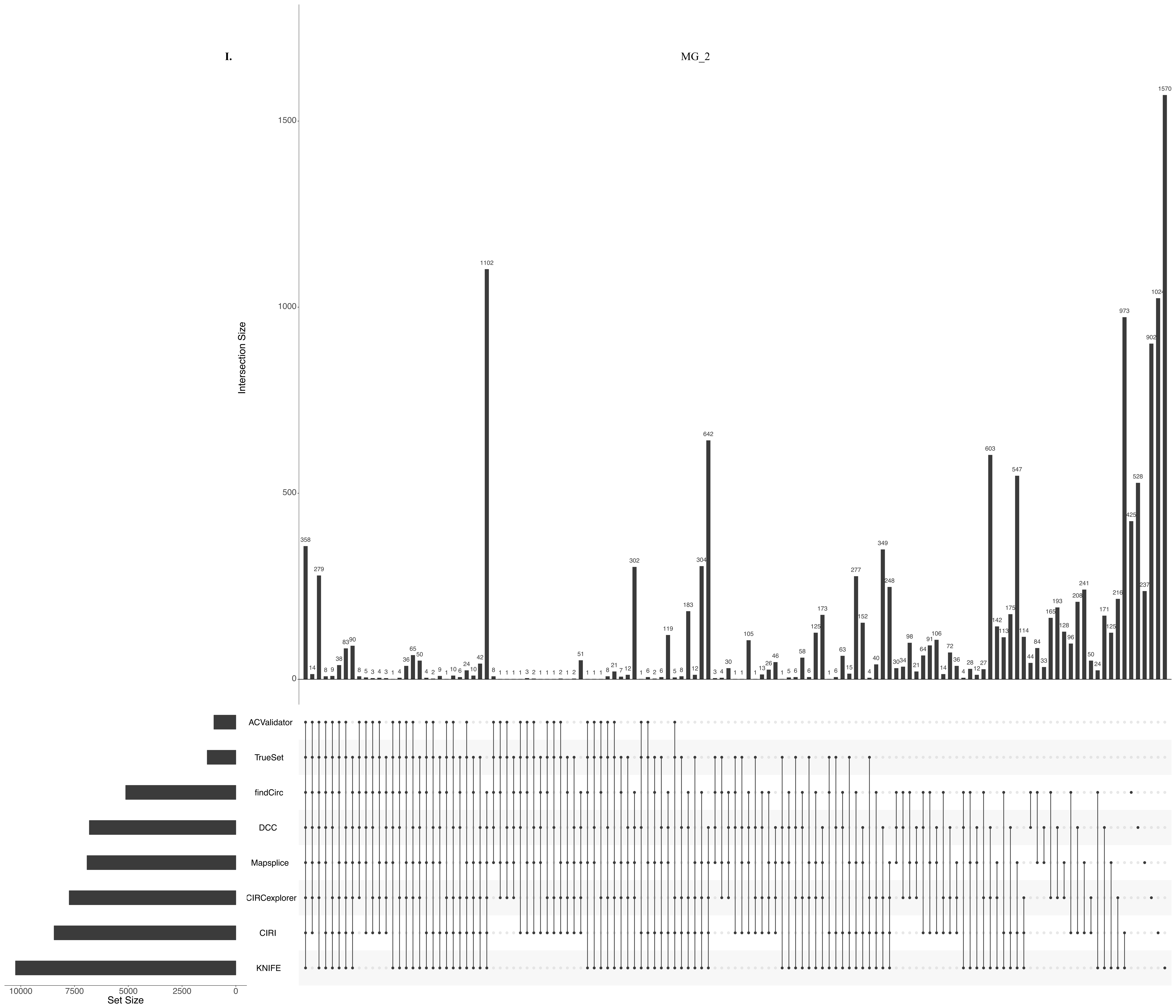
I.
MG_2
Intersection Size
ACValidator
TrueSet
findCirc
DCC
Mapsplice
CIRCexplorer
CIRI
KNIFE
Set Size

J.

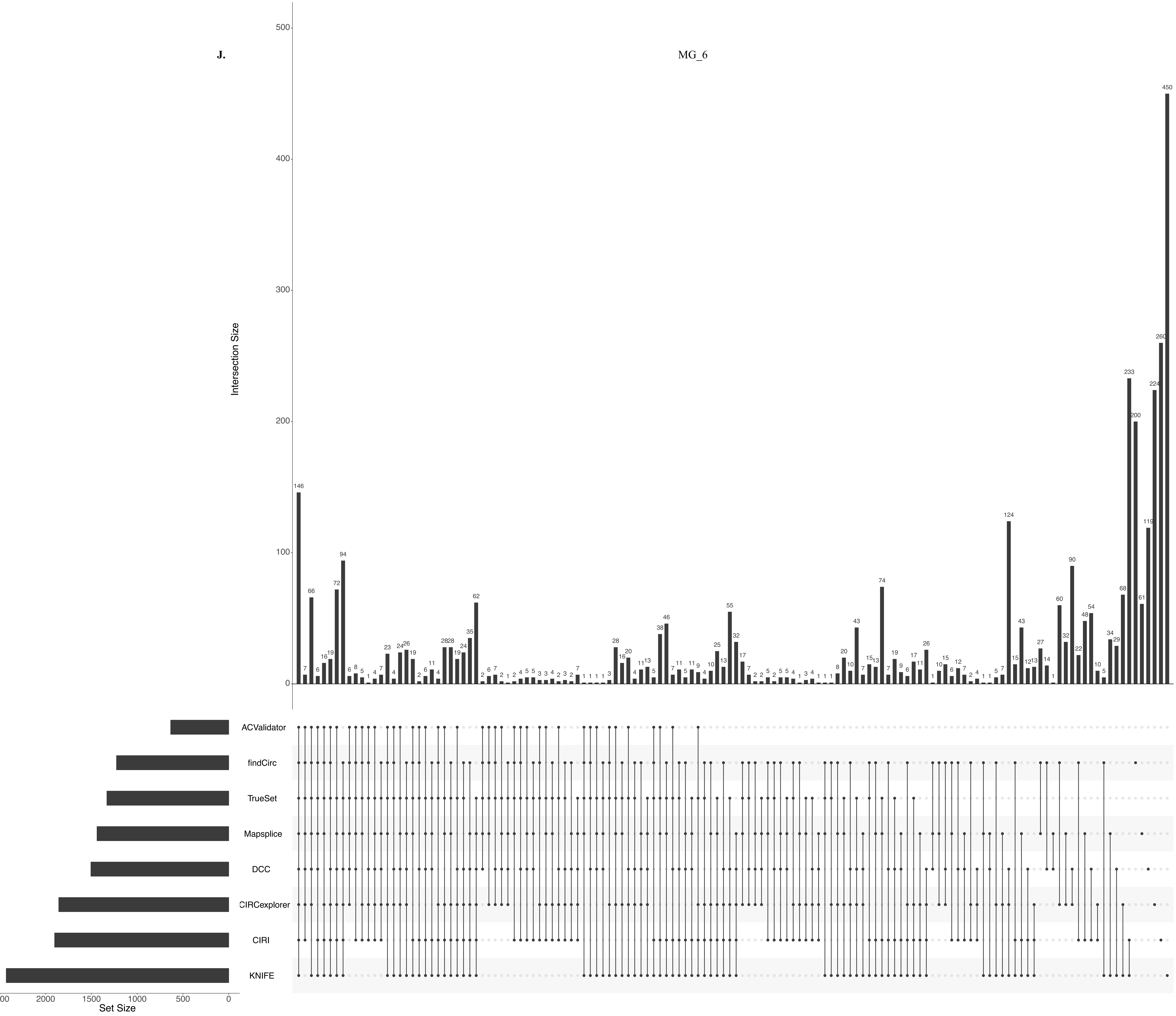
MG_6
Intersection Size
ACValidator
findCirc
TrueSet
Mapsplice
DCC
CIRCexplorer
CIRI
KNIFE
Set Size

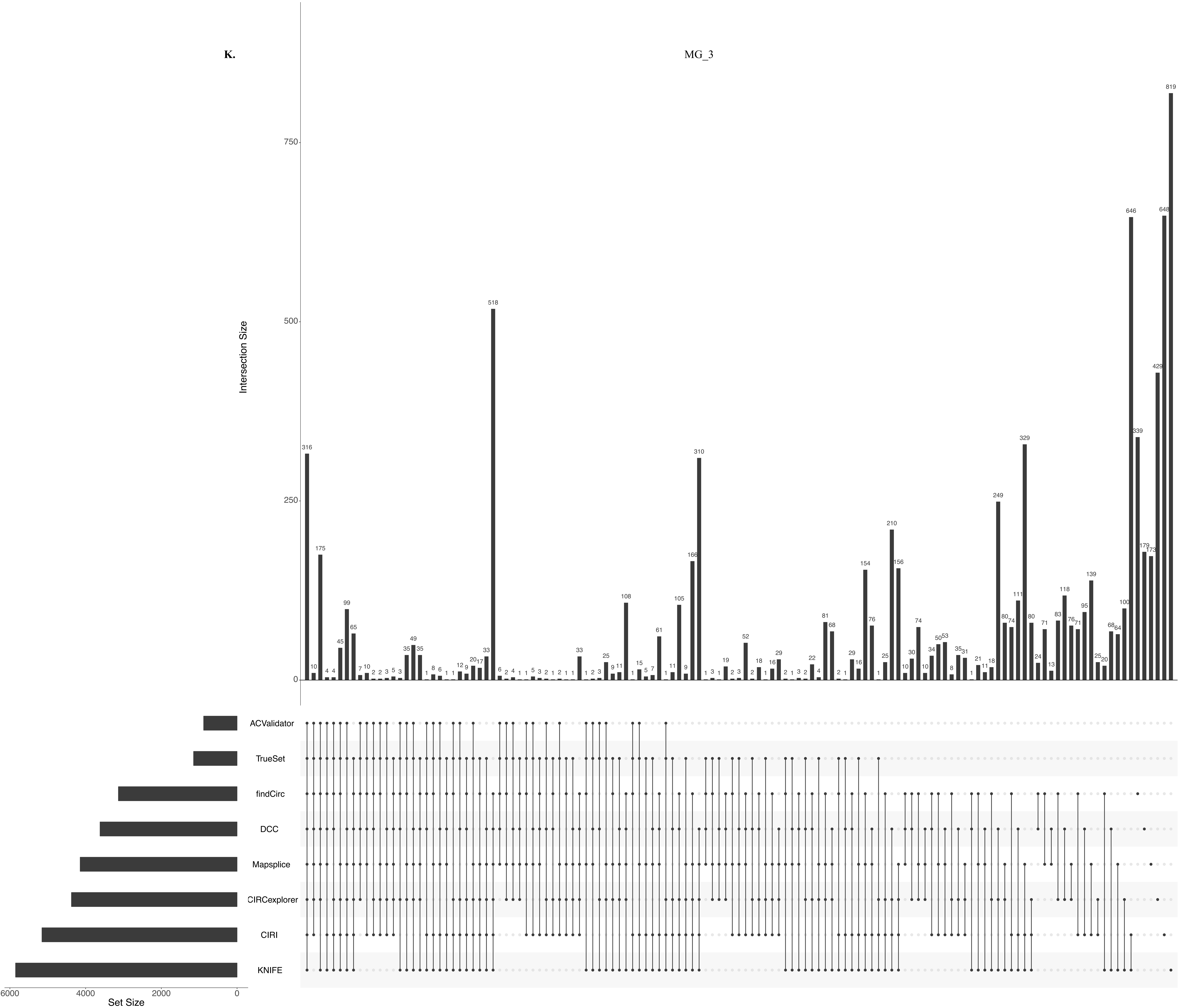
K.
MG_3
Intersection Size
Set Size
ACValidator
TrueSet
findCirc
DCC
Mapsplice
CIRCexplorer
CIRI
KNIFE

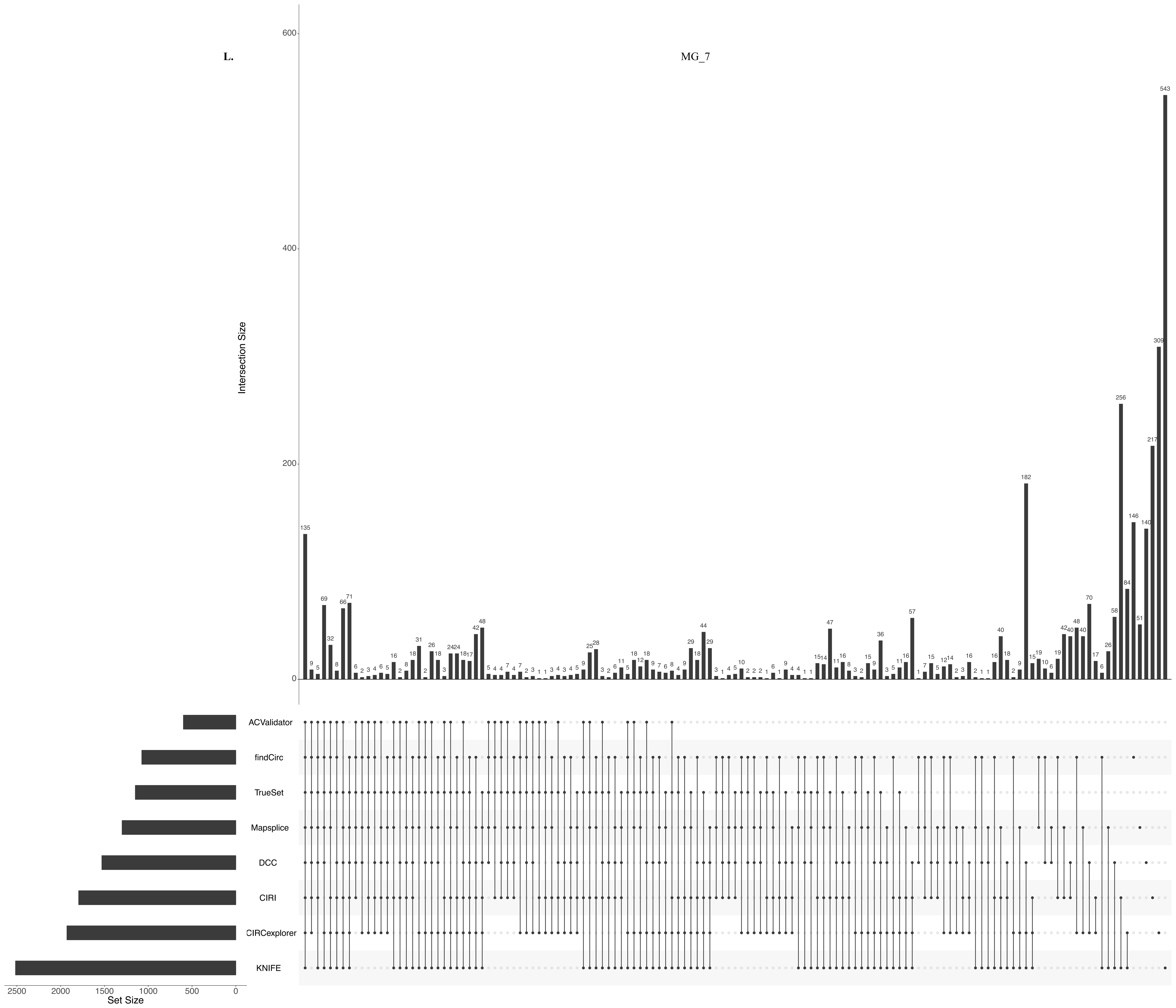
L.
MG_7
Intersection Size
Set Size
ACValidator
findCirc
TrueSet
Mapsplice
DCC
CIRI
CIRCexplorer
KNIFE

Supplementary Figure 2. Upset plot of circRNAs detected in experimental datasets using different approaches. Intersection of circRNAs called by each of the six existing tools (labeled by tool name), circRNAs validated by ACValidator when using low stringency criteria and window size = 2 * insert size (labeled “ACValidator”), and our defined set of true circRNAs (labeled “TrueSet”). We considered those candidates that were called by at least three of the six tools in both the treated and non-treated samples, and not depleted following RNase R enrichment, as true circRNAs. Panels A-L represent the upset plot for each dataset.
