# Supplementary material for: ACValidator: A novel assembly-based approach for in silico verification of circular RNAs
Source: Biol Methods Protoc. 2020 Aug 10;5(1):bpaa010. doi: 10.1093/biomethods/bpaa010 (PMC7415914; doi:10.1093/biomethods/bpaa010)
Supplement: bpaa010_Supplementary_Data [file bpaa010_supplementary_data.zip › S3_Fig_v2.pdf]

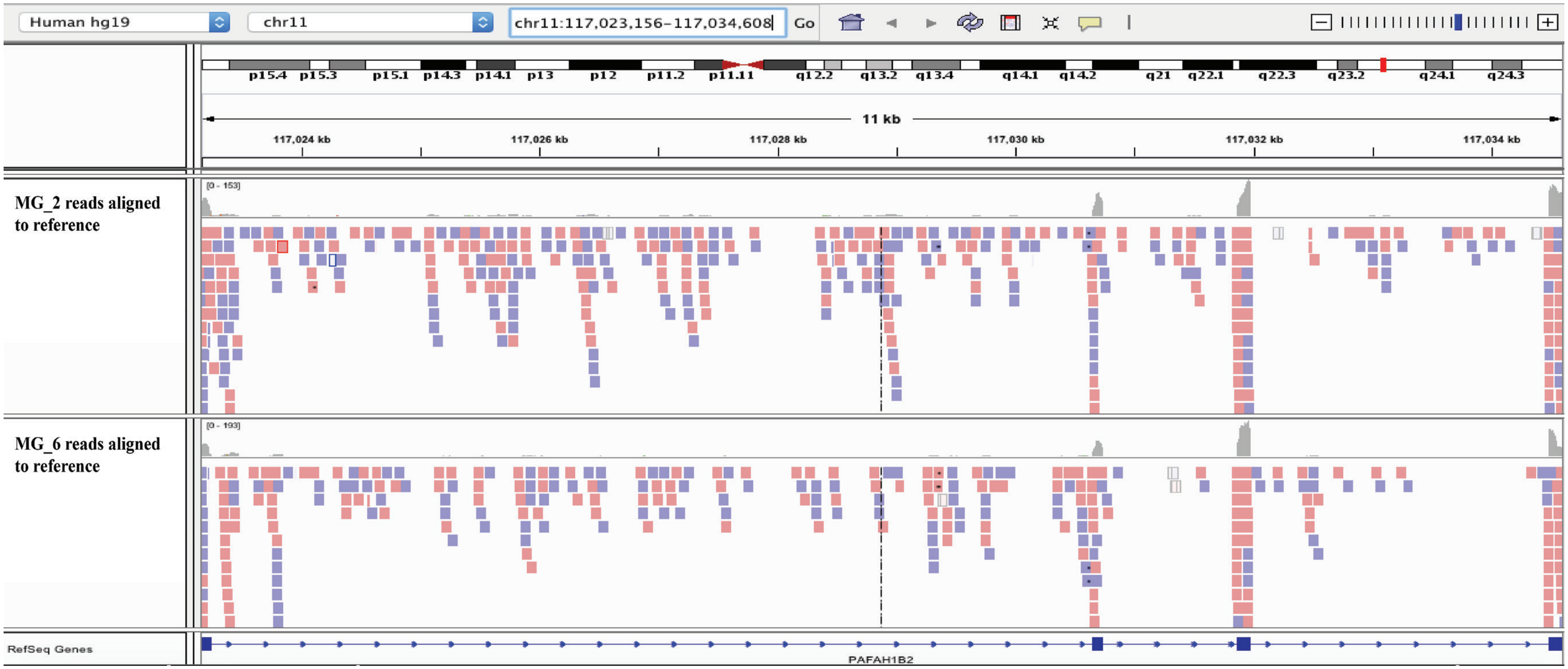


**Supplementary Figure 3**. Reads from the RNase R-treated (top panel) and non-treated (bottom panel) MG samples, MG_2 and MG_6 respectively, aligned to the human reference genome (hg19).
