# Supplementary material for: ACValidator: A novel assembly-based approach for in silico verification of circular RNAs
Source: Biol Methods Protoc. 2020 Aug 10;5(1):bpaa010. doi: 10.1093/biomethods/bpaa010 (PMC7415914; doi:10.1093/biomethods/bpaa010)
Supplement: bpaa010_Supplementary_Data [file bpaa010_supplementary_data.zip › S4_Fig_v2.pdf]

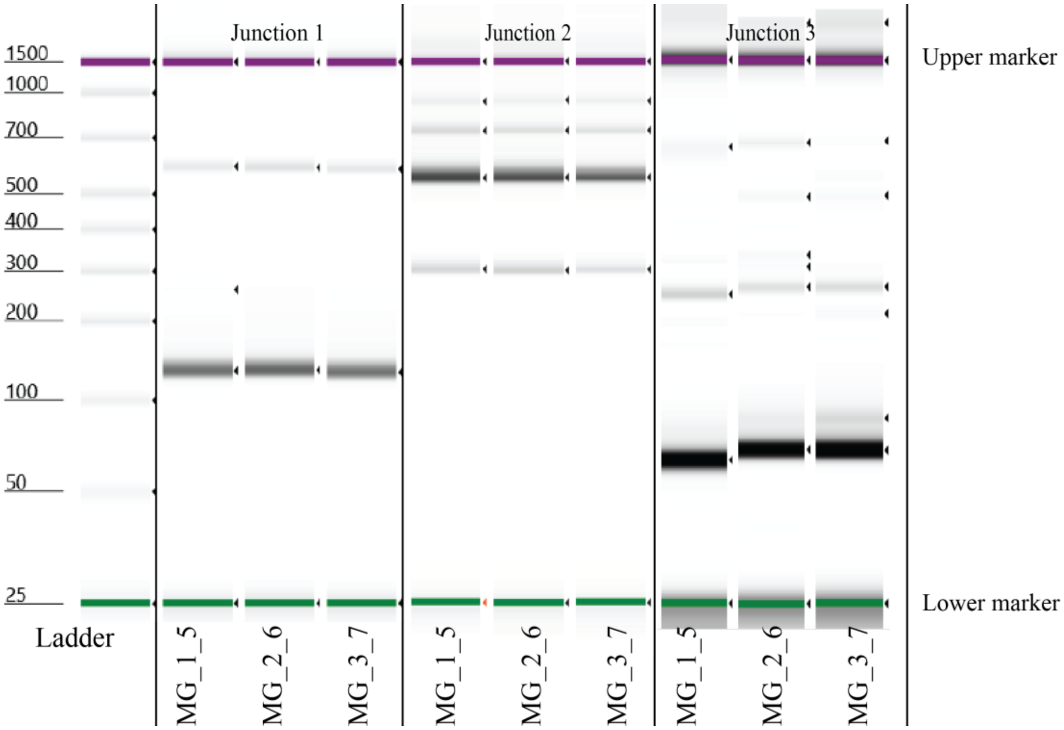

Supplementary Figure 4. A. PCR validation of selected highly expressed circRNA candidates. Six highly expressed circRNA candidates (average SRPBM > 650) that were validated by ACValidator and detected by all the six algorithms were selected for validation. Among these, ACValidator was able to validate chr10:116,879,948-116,931,050 in two of the three samples, and chr9:113,734,352-113,735,838, chr8:37,623,043-37,623,873 and chr5:38,523,520-38,530,768 in all three samples. For the remaining two candidates, we observed evidence of validation but because differently sized PCR products were generated, we could not determine the exact product size. Left panel: chr5:38,523,520-38,530,768 (junction 1), expected product size: 130bp; middle panel: chr10:116,879,948-116,931,050 (junction 2), expected product size: 679bp; right panel: chr9:113,734,352-113,735,838 (junction 3), expected product size: 76bp.

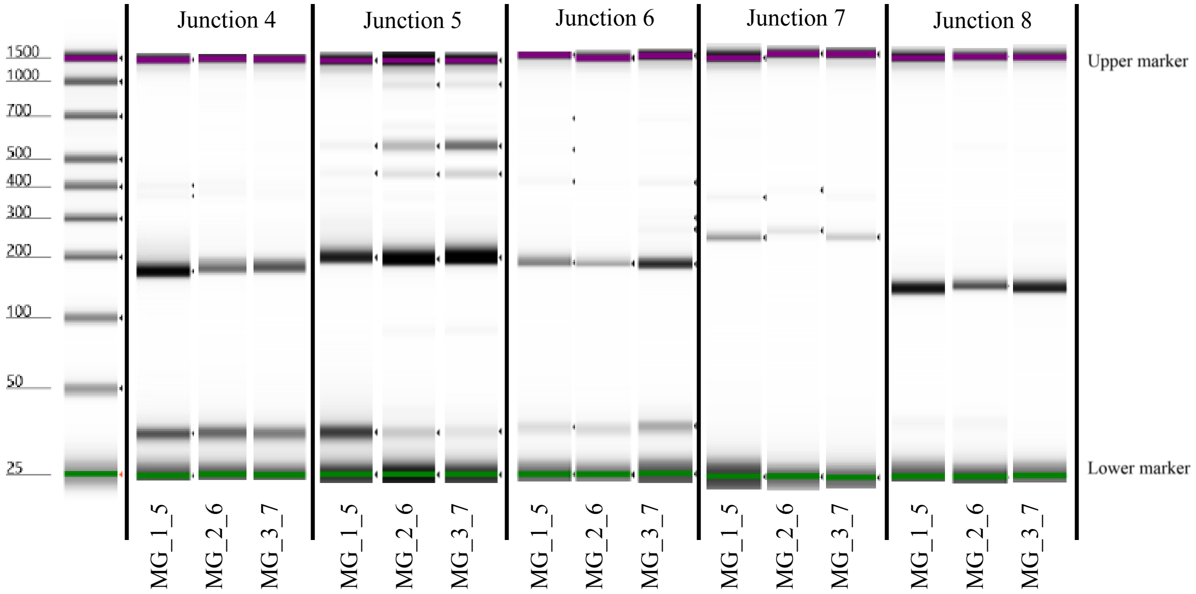

B. PCR validation of selected medium expressed circRNAs candidates. Four medium expressed circRNAs candidates (600 > average SRPBM > 300) that were validated by ACValidator and that were detected by three of six algorithms were selected for validation. All circRNAs junctions were validated across the three untreated MG samples. Example Agilent TapeStation gel traces are shown. Validated circRNAs include chr5:10,415,599-10,417,516 (junction 4), expected product size: 183bp; chr7:8,257,934-8,275,635 (junction 5), expected product size: 208bp; chr5:64,084,777-64,100,213 (junction 6), expected product size: 195bp; chr4:56,277,780-56,284,152 (junction 7), expected product size: 248bp; and chr8:37,623,043-37,623,873 (junction 8), expected product size: 154bp.

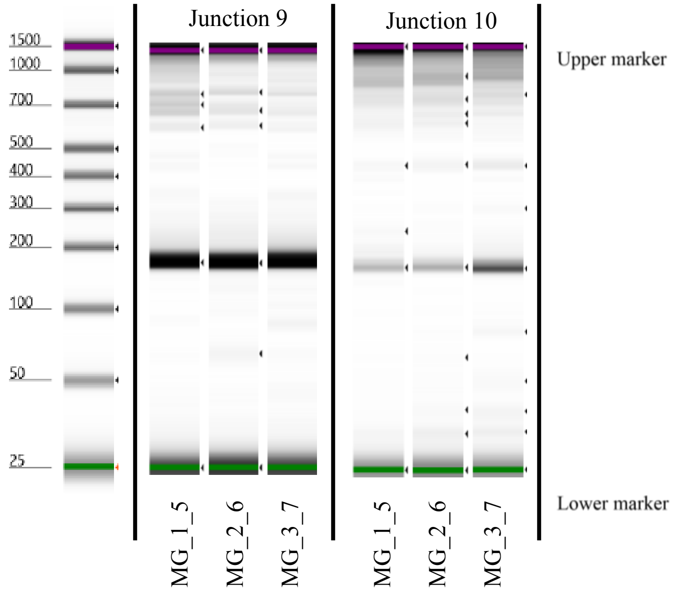

C. PCR validation of selected lowly expressed circRNAs candidates. Two lowly expressed circRNAs candidates (average SRPBM < 90) that were validated by ACValidator and that were detected by three of six algorithms were selected for validation. Both circRNAs junctions were validated across the three untreated MG samples. Agilent TapeStation gel traces are shown. Validated circRNAs include chr15:93,540,186-93,545,547 (junction 9), expected product size: 182bp; and chr3:3,178,943-3,186,394 (junction 10), expected product size: 172bp.
